# Synchronising institutions and value systems: A model of opinion dynamics mediated by proportional representation

**DOI:** 10.1371/journal.pone.0257525

**Published:** 2021-09-28

**Authors:** Jose Segovia-Martin, Monica Tamariz

**Affiliations:** 1 Institut des Systèmes Complexes de Paris Île-de-France (ISC-PIF), French National Centre for Scientific Research (CNRS), Paris, France; 2 Department of Psychology, Heriot-Watt University, Edinburgh, United Kingdom; Central European University, HUNGARY

## Abstract

Individuals increasingly participate in online platforms where they copy, share and form they opinions. Social interactions in these platforms are mediated by digital institutions, which dictate algorithms that in turn affect how users form and evolve their opinions. In this work, we examine the conditions under which convergence on shared opinions can be obtained in a social network where connected agents repeatedly update their normalised cardinal preferences (i.e. value systems) under the influence of a non-constant reflexive signal (i.e. institution) that aggregates populations’ information using a proportional representation rule. We analyse the impact of institutions that aggregate (i) expressed opinions (i.e. opinion-aggregation institutions), and (ii) cardinal preferences (i.e. value-aggregation institutions). We find that, in certain regions of the parameter space, moderate institutional influence can lead to moderate consensus and strong institutional influence can lead to polarisation. In our randomised network, local coordination alone in the total absence of institutions does not lead to convergence on shared opinions, but very low levels of institutional influence are sufficient to generate a feedback loop that favours global conventions. We also show that opinion-aggregation may act as a catalyst for value change and convergence. When applied to digital institutions, we show that the best mechanism to avoid extremism is to increase the initial diversity of the value systems in the population.

## Introduction

Opinions and beliefs are the product of socially transmitted information and follow evolutionary dynamics [1, 2]. The digital era has led to the emergence of online platforms, where content is created under the influence of platform algorithms, which are integrated systems of rules that structure social interactions. These platform algorithms act as digital institutions that aggregate information and transmit such content back to their users [3–5].

Recently, academic interest has increased around how these platforms affect opinion dynamics. There are many reasons: connectivity between online platform users is greater than in offline communication, social learning is mediated by digital institutions, and more and more people form their opinions through social media [6–8]. Indeed, there is work on (and concerns about) how social media affects consensus [9, 10], influence [11–13], polarization [14–16], extremism [17, 18] and disagreement [19, 20].

Online social learning is shaped by key information aggregated by social media platforms. These platforms have their own interests and they use their algorithms, based on users’ behaviour, to choose content, manipulate it and transmit it back to their users. For example, some social media platforms aggregate information and display content tailored to each user, while others display the most successful or mainstream content. Among the many systems for aggregating information, a particular example is the principle of proportional representation (PR). PR characterises aggregation systems whose result proportionately reflects the information obtained from the population. For example, if a digital platform were to aggregate expressed opinions using a PR algorithm, the information shown to users would proportionally represent the opinions expressed by the population. If a digital platform were to aggregate cardinal preferences (i.e. relative preference intensities) over opinions using cardinal PR, the information shown to users would proportionally reflect the class of proportionally rated opinions. PR is designed to promote close correspondence between the proportion of support of one group of interest in the population and the proportion of such group of interest in a governing body (e.g. in a digital institution). As opposed to customised recommendations or majority-based rules, under PR each and every opinion or preference contributes to the final result. In this research we will explore opinion dynamics mediated by PR.

Proportional representation aims to solve the problem of unfairness of majority rules where minority options are usually underrepresented or not represented at all [21, 22]. Although PR systems are usually intended to improve electoral systems, with social media platforms we also have something similar to “en bloc voting”. Reddit posts visibility based on relevance, Google searches, Facebook likes or Twitter retweets are examples mediated by aggregation algorithms that use a combination of majority rules and personalised recommendations. The development of systems that are able to control for asymmetric information in the social landscape seem to be crucial for the development of platforms that reconstruct opinion dynamics to represent the whole political community. In this regard, projects such as *Politoscope* [6] are proposing an integrated methodology for the aggregation, reconstruction and analysis of the spectrum of political orientations representing a country’s electorate, which might help develop new and more diverse forms of communication in social networks.

Despite the alleged benefits of PR, to the best of our knowledge, there are no social media platforms whose opinion dynamics are mediated by a digital institution that aggregates information according to the principles of PR. Thus, we do not know how opinion dynamics in such platform might evolve. In this paper we examine precisely how opinion dynamics evolve in a platform mediated by an institution that aggregates information using the principle of proportional representation and shows it back to the agents. In particular, we look at the conditions under which convergence on shared opinions can be obtained under opinion-aggregation institutions (i.e. a method that aggregates the actual agents’ expressions irrespective of the underling system of preferences) and value-aggregation institutions (i.e. a method that aggregates agents’ cardinal (utility-based) preferences or “value systems” irrespective of the agents’ actual opinions). Based on existing knowledge of previous theories on decision making and institutional rational choice (e.g. [23–25]), we expect the aggregation of individual inputs (i.e. opinions and preferences) into collective outputs (i.e. institutions and the overall diversity of opinions) to affect the dynamics of opinions and the emergence of consensus under proportional representation.

We develop an agent-based model (ABM) that simulates opinion dynamics in a social media platform where connected agents repeatedly update their preferences under the influence of PR institutions. We consider a population of agents that have to observe and transmit opinions to their neighbours at each time step. In a standard social learning model, information is transmitted from agent to agent, and agents update their prior hypotheses with the new information at each time step. In our model, we add the influence of a digital institution (platform algorithm) that aggregates information following the principle of PR and presents such information back to the population. This iterative process is modelled as a stochastic process that describes a sequence of possible opinions, in which the probability of each agent’s opinion depends only on the state such agent attained in the previous time step. At each time step, the status of each agent is updated according to the influence of the institution, its own value system and its own opinion. Institutions are also updated at each time step according to the proportional representation of population’s opinions (in the case of opinion-aggregating institutions) or of population’s “value systems” (in the case of value-aggregating institutions). We examine the behaviour of our model under different conditions of biased learning and population homogeneity (see Section Model). The next subsection places our study in the context of recent models/work.

### Modelling opinion dynamics in social networks

A growing body of research is devoted to modelling opinion dynamics in social networks. Most approaches in this field are based on the DeGroot model [26], where agents update their beliefs as a weighted average of the beliefs of their neighbors, with weights given by the degree of “trust” they place on those neighbors. Building on DeGroot’s model, a huge number of variants of the update rule have been implemented to facilitate the analyses of persistent disagreement, polarisation and extremism in social networks [27–29]. In Anunrojwong et al.’s model [30] agents repeatedly update their opinions based on their neighbors’ beliefs and the information shown by an institution (i.e. a digital platform) that makes personalized recommendations. Broadly speaking, Anunrojwong et al. found that agents’ opinions became more extreme when the platform’s influence is very weak or very strong. On the contrary, intermediate influence led to moderate persistent disagreement, where agents disagree but with low extremism. We look closely at this model but also move away from it in two important aspects. First, instead of modelling using differential equations, we build on a tradition of agent-based models of cultural evolution that aims at representing the micro-level behavior of individual agents [2, 31, 39, 40]. Our approach to modelling multilevel selection is endogenous, as our institutional structures and group interactions emerge from individual characteristics and processes. This endogenous approach will permit us to detect the strength of selection at any organizational level (i.e. individual and institutional). Second, previous models simulating platforms that make personalised recommendations do not explain opinion dynamics on platforms under PR. In this study, we focus precisely on institutions that aggregate and transmit information using the principles of proportional representation (PR).

Other studies have used a mean-field feedback rule (i.e. a rule that uses a single average to approximate the effect of all individuals on any given individual) to model the influence of an exogenous signal that is constant and transmitted to either specific agents (as in Hegselmann & Krause [32]), or using particular distributions of exogenous opinions (e.g., as in Kolarijani et al. [33]). In Hegselmann & Krause’s study, a bounded confidence model is proposed, where none of the agents influence the signal, which has a constant value. However, the signal has a direct impact on agents’ opinions. Hegselmann & Krause analysed the effect of different intensities of the exogenous signal and found that, surprisingly, the more intensive signal may have less effect. In Kolarijani et al., they considered a model for opinion dynamics under exogenous influence of static opinions and environmental noise. In this model, noise represents the uncertainties [34] or the effect of free will [35] in agents’ opinions. They showed that the number of different clusters of opinion decreases as the level of noise increases. That is, the effect of the exogenous signal is restrained in systems with high level of noise. In our model, the degree of “uncertainty” is modelled by the implementation of cardinal probability distributions of an uncertain weight of opinions. Each agent’s normalised cardinal vector expresses agent’s beliefs before being exposed to new opinions. We call these cardinal vectors “value systems”. We test whether, as in Kolarijani et al., high levels of value dispersion in agents’ value system also restrain the effect of institutional influence.

In contrast to these previous studies (whose external signal implementations may not be realistic given the endogenous nature of institution building [36]), we now propose an agent-based model of opinion dynamics based on a simple (arithmetic) non-constant mean-field feedback rule (called “institutional influence”). Unlike Hegselmann & Krause [32] or Kolarijani et al. [33], our feedback is not constant, but evolves as a reflexive signal (i.e. a function that transmits information bi-directionally, from the agents to the institution and from the institution to the agents) aggregating the opinion of all agents using a rule of proportional representation (PR). To our knowledge, this notion of “synchronizing institution” which endogenously aggregates opinions is novel within the agent-based modelling literature on opinion formation and polarization emergence. The endogenous generation of institutions by a proportional aggregation rule could have drastic effects on the long-term behaviour of opinions, substantially modifying the equilibrium of opinions when compared with models based on exogenous influence of static opinions. This motivates examination of the system proposed in the present study. In the present study we compare our results against these previous models and will analyse the extent to which our model exhibits a different behavior. In particular, we use a multi-parametric model to evaluate how the intensity of institutional influence, agents’ value systems, population homogeneity and biased learning affect opinion dynamics.

Our model is also related to a number of models that explore the spread of cultural variants in social learning scenarios where agents are endowed with cognitive biases that affect the likelihood of adopting a given variant [37–40]. These models implement a *content-based bias*, also termed direct bias by Boyd and Richerson [2], which in our case refers to individual’s sensitivity for opinion’s value. As a consequence of this, the more sensitive an agent is and the more valuable an opinion is to that same agent, the more likely it is that such opinion will be adopted by the agent.

Content-biased models generally assume that all agents in the population assign the same value to the different cultural variants. A population of agents with high content bias, for instance, leads to a reduction in cultural diversity [39–41]. However, in real life not everyone shares the same values, and different sub-populations may develop dissimilar *value systems* (e.g. opposed interests). The effects of different value systems on the diversity of cultural variants have been explored in the past. For example, Axelrod’s model of dissemination of culture [42] was based on the assumption that people are more likely to interact with others who share the same cultural variants, and this in turn tends to increase the number of variants they share. These mechanisms, named *homophily* and *influence*, are prominent explanations for the persistence of cultural diversity [42]. When combined with dynamic co-evolving networks, they can lead to stable cultural diversity in the face of cultural drift [43]. Building on [42], researchers have found that the dynamics of cultural change and cultural diversity are affected by globalization [44], technological innovation [45], differences of opinion [46, 47], mass media [48, 49], political institutions [50] and cultural drift [51], among others.

It is important to note, however, that our model does not in any way allow us to discern which information is true and which information is false. In other words, the simulations that we present permit us to represent the state of diversity of opinions in a micro-society, but there are no opinions that are truer than others. Therefore, more work is needed to explore whether the selected opinions represent maladaptations or misinformation.

### Value systems and their distribution in the population

In decision theory, preferences and options are the two central concepts. Roughly speaking, when we say that an agent “prefers” the “option” A over B we mean that the agent takes A to be more useful or desirable than B. For example, if we consider the spectrum of ‘political ideologies’, somebody may value ideology X more than Y or Z, while somebody else may prefer Y and Z equally, and disprefer X. The numerical representation of preference orderings is crucial for the formalisation of the decision-making process. These numerical measures are traditionally known as utility functions. The two main types of utility function used in decision theory are ordinal utility function (mostly used in consumer theory under certainty) and the more information-rich interval-valued (or cardinal) utility function (used in game theory under uncertainty). These labels have traditionally been used to denote sets of rational preferences, however, as Bayesian decision theory shows, there is an inevitable connection between rational preferences and rational beliefs. However, many prefer not to equate belief with preferences over options. At the heart of this unresolved question lies the epistemological problem of the origins of value and the foundations of the expected utility theory [52].

In our simulations we will consider that given a set of opinions, each agent is endowed with a cardinal utility vector transformed into a distribution of preferences over the set of opinions in the population. For the sake of simplicity, and to avoid adopting a strong epistemological position on the origin of preferences or beliefs, we will call this vector simply *value system*. In the system we simulate, value systems can change. For example, we are sensitive to partner’s opinions, institutional norms or economic pressures, which can change our ideas and behaviour in pursuit of a better adaptation to the environment. Each value in an agent’s value system corresponds to the agent’s preference for a particular opinion.

Because cumulative changes in agent’s value systems produce opinions where future states of the opinions depend on the past states of that opinion, it is the kind of process that generates path dependence: that is, network interactions can impose structural and situational constraints that influence agents’ opinions. Indeed, historical chains of cultural variants, as may be the case with opinion acquisition, are linked through patterns of cultural transmission where agents are assumed to suffer from historical dependencies [40, 53–55]. That is, agents’ opinion choice at each time step is constrained by the opinions agents have chosen in their recent past. In our model, we adopt this iterative modelling approach: we consider that the value that an agent assigns to a future opinion is a function of three well-established sources of variation: current opinion, experience and institutional influence.

Individual value systems can be more or less hegemonic. For instance, one opinion may be preferred over all the others (e.g. there is only one dominant political party), or two opinions may be equally preferred over all the others (e.g. two political candidates, say one Democrat and one Republican, competing for victory in a tight US presidential election), or all opinions may be equally likely to be produced (e.g. diverse people giving their first opinion on an unknown new topic).

Individuals in a society may also have common or opposing interests [56, 57]. In this respect, populations can be either *homogeneous*, when all agents share the same variant inventory and the same value system (e.g. the population of mathematicians, who share the same mathematical conventions, and assign the same value, or meaning, to each of them), or *heterogeneous*, when agents share the same variant inventory but there are two or more value systems (e.g. agents from different groups such as employers and employees may assign different value to variants such as *flexibility* and *precariousness* even if they refer to the same payoff matrix; and the value of vaccines is very different for anti-vaxxers and the rest of the population). The reader should not confuse *heterogeneity* of value systems (number of independent *S*), as used in the current paper, with the *heterogeneity* of the population (number of traits, *q*) as defined in related literature [43].

### Institutions

Institutions are “integrated systems of rules that structure social interactions” [58, p. 501], or norms and conventions that give durable structure to social interactions within a population [59]. They are not only mere providers of goods and services, they also influence the evolution of values, tastes, and personalities [36, 60, 61].

Social media scholars have recently begun to use institutional theory as a framework to analyse the role of algorithms in social media platforms. In the context of digital platforms where interactions between users are mediated by algorithms that aggregate information hierarchically and structure social interactions, algorithmically-driven search and recommendation systems can be considered an instance of the notion of institution [3–5].

#### Plurality vs. cardinal systems

Just as elected governments in democratic countries emerge from their voting system, digital institutions on a social platform can emerge from the opinion aggregation system. Plurality voting and cardinal voting are the most commonly used systems to aggregate opinion. In plurality voting, each agent is allowed to choose only one option, while in cardinal voting each agent assigns an independent evaluation to each option. Plurality and cardinal systems give rise to different forms of preference expression. In the former, only the expression of the preferred option is known; in the latter, the magnitudes of preferences over options are known. Cardinal utility assumes that absolute satisfaction levels exist.

The analogy can be applied to the social scenario we simulate, where the set of options to be chosen is a set of opinions to be expressed. In our model, each agent expresses a single opinion in each round and is also endowed with a cardinal preference vector (or value system). This allows us to create and compare two types of emerging institutions: Institutions that aggregate the opinions expressed by the agents (opinion-aggregating institutions) and institutions that aggregate the cardinal preferences (or value systems) of the agents (value-aggregating institutions).

#### Opinion-aggregating vs. value-aggregating institutions

We consider two institutions according to the type of information the algorithm aggregates: opinion-aggregating institutions and value-aggregating institutions.

(i) Opinion-aggregating institutions: Consider the set of opinions over all agents as the set of preferred choices. Then, assuming that agents are treated symmetrically (i.e. in each simulation, all agents have the same biases and the opinion of all agents have the same weight), the combination of agents’ choices can be interpreted as a frequency distribution of opinions in the population (e.g. [62]). If each agent expresses a single opinion and *f*_*x*_ is the relative frequency of opinion *x* in the population, then, under strict proportional representation, *f*_*x*_ will correspond to the weight opinion *x* will have in the emergent institution. That is, in an opinion-aggregating institution, each opinion *x* will have an associated weight calculated as the relative frequency with which *x* is expressed by all individual agents. Note that this is a non-utilitarian method, since the agents have not assigned value or utility to each one of the opinions, but have simply expressed (or chosen) them. That is, the resulting institution does not bases decision on agents’ direct valuation of opinions. On the contrary, the resulting model can be thought of as an approximation of a platform that aims to use the actual expressions of individuals irrespective of their underlying value systems.
  Given a vector of relative frequencies of opinions generated with an opinion-aggregating institution, one could use a first-past-the-post rule to choose the opinion with the highest frequency and display such opinion to the users of the platform. This system can also be referred to as *bloc voting*, and is widely used by digital platforms to make specific content more visible. It is also used in many electoral processes. On the contrary, under a proportional representation rule, users’ opinions are reflected proportionately in the social media platform. In the present study, we are interested in investigating how opinion dynamics evolve under the influence of this type of symmetrical proportional representation.
(ii) Value-aggregating institutions: We also consider the existence of an omniscient institution that is able to optimally aggregate agents’ value systems. The idea that agents’ cardinal (utility-based) preferences are directly observable, measurable and comparable has widely been used in social choice theory and behavioral economics. A model of such value-aggregating institution can be viewed as an approximation for a social media digital algorithm that aims to identify and exploit conditions under which agents’ real underlying cognitive states are more important than mere momentary productions (e.g. digital platforms tools deploy machine learning, statistics, and natural language processing techniques to automate sentiment analysis with the aim of extracting subjective information from social media users and filter it to make recommendations). Similar idealised approaches, where institutions are assumed to be able to aggregate unavailable information, have traditionally been used by economists to account for institutions pursuing the public interest, or specify models that describe an optimal outcome, such as an optimal allocation of resources [63–65]. Different instances of this notion have also been used in social choice theory [66] and social network studies to describe omniscient data observation [67].
  Given a vector of values generated within the framework of a value-aggregating institution, one could use for example a highest median rule to select the opinion with the highest median grade and display it to users (e.g. majority judgment). However, in this study we are interested in the specific opinion dynamics in platforms using proportional representation so we will use an arithmetic mean (with equal weights for all the agents) to aggregate the value system vectors. This method assumes that the platform recommendation should be a compromise between vectors. This aggregation rule has found use in the Analytic Hierarchy Process (AHP) literature; e.g, [68].

#### Institutional influence

In the present study we will use the term *institutional influence* to denote the effort on the part of a digital institution to favor a particular value system, which may lead to changes in the value systems of individuals. Real-life examples of institutional influence include the approval or condemnation of particular usages of grammar and vocabulary by national linguistic academies, peer pressure to conform to the behaviour of a social group, and the prescription of a moral code that encourages people to do what is right and not to do what is wrong according to that code. In the simulations presented below, the value system reinforced by institutions emerges from the proportional aggregation of agents’ preferences. This models the potential opinion dynamics in a platform mediated by PR, in scenarios with null (agents are not compliant with institutional recommendations), intermediate and strong (agents’ are fully compliant) influence.

Theories of collective behavior suggest that institutional mechanisms (e.g. the introduction of collective incentives) can reduce cultural diversity by facilitating the formation of social conventions [69–72]. In contrast, social evolutionary theories have suggested that social conventions can emerge spontaneously without social institutions in place to guide the process [73, 74], and a number of studies have successfully tested this and related hypotheses in the lab [75, 76]. Our model will test whether convergence conditions exist in the absence and near-absence of institutional incentives. As for the type of consensus that is reached under different intensities of institutional influence in social media platforms, when platforms make personalised recommendations, strong influence increases polarisation in extreme opinions, intermediate influence decreases extremism, and weak institutions increase the likelihood of a consensus on extreme opinions [30]. A number of bounded confidence models suggest that greater dispersion, uncertainty or noise in agents’ opinions could reduce extremism [30, 33], while others suggest that a greater number of radicals could lead to less radicalization [32]. We will compare these models and our simulations and propose directions for future research.

To sum up, our goal in this study is to examine opinion convergence dynamics in populations of agents whose interactions are influenced by PR institutions. We will use a one-dimensional (left-right) opinion spectrum to explore how the interaction between institutional influence, initial value systems in the population and biased learning affects the emergence of conventions and the long-term stabilised ratios of competing opinions (i.e. the type of consensus, polarisation and diversity of opinions when the frequency of opinions stabilises in the model). We will compare two types of aggregation rules according to the nature of the aggregated information: a non-utilitarian (opinion-aggregation) and a utilitarian (value-aggregation) algorithm. Our multi-selection ABM models emergent institutions by aggregating opinions and value systems. The institutions’ rules are updated with changing opinions and values over time in an iterative process. Our results speak to questions such as how opinion diversity can be maintained and how convergence on shared opinions can be obtained in such a system. In this respect, we will also compare our results to those of simulations of different types of institutions.

## Model

We consider a micro-society of agents interacting on a social media platform governed by a digital institution that aggregates information using the principles of proportional representation. Each agent is characterized by a number of state variables as described in section Model parameters and equations. The micro-society initially contains *N* agents, who pair-up and interact for a number of rounds (*R*). Each interaction consists of an exchange of opinions selected from an initial pool of variants (*X*).

**Fig 1:**
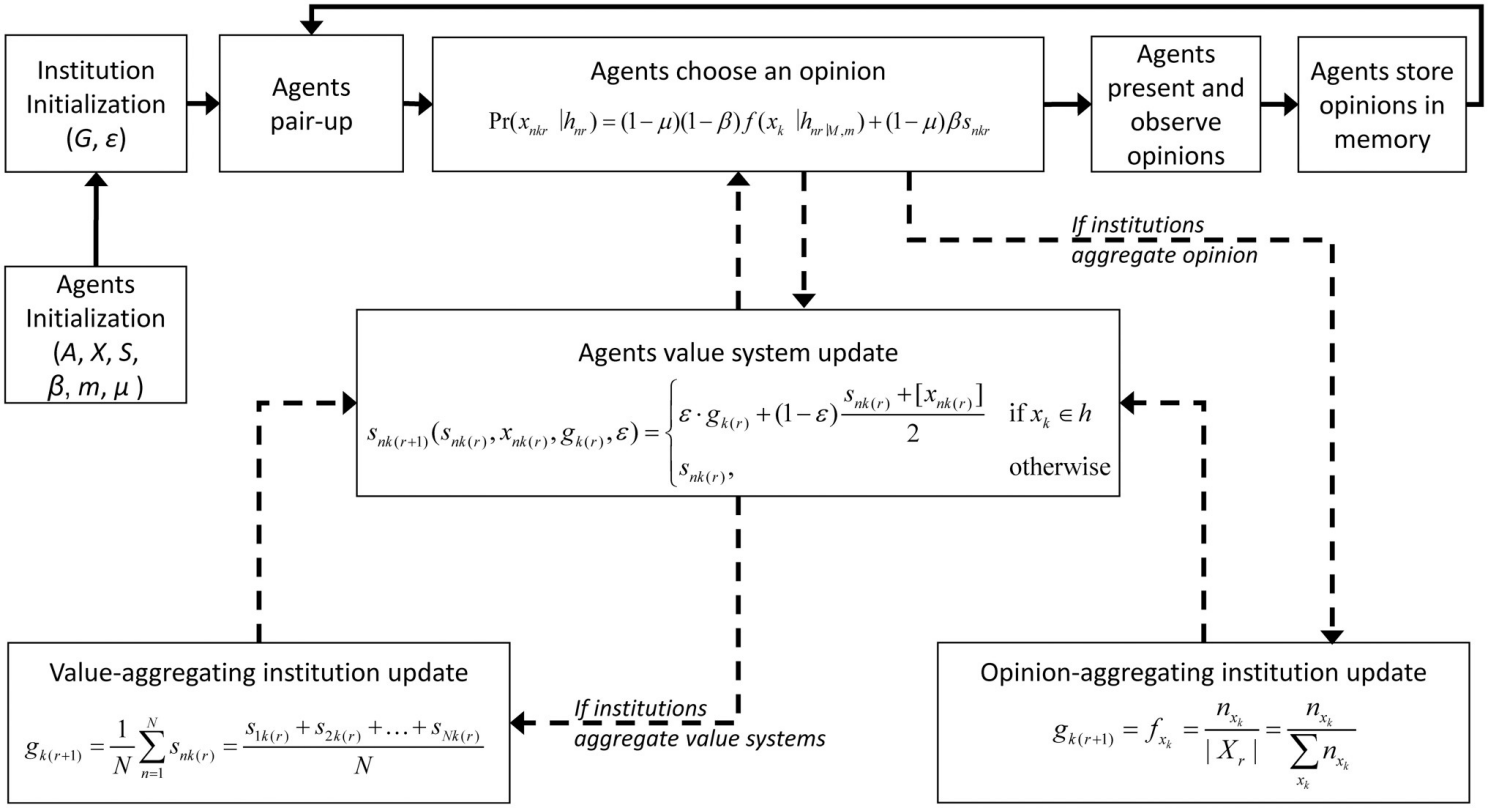
Flowchart depicting relevant activity during one round. Arrow direction represents the time-flow of events. Plain lines represent transition from one event to the following one. Dashed lines represent new data that is used to update the prior information of agents and institutions, affecting agents’ opinion choice over time. We simulated two institutions according to the type of information they aggregate: value-aggregating institutions and opinion-aggregating institutions. Parameters and symbols can be found in Table 1.

Agent pairings are scheduled using a method that takes the sequence of agents of the population after each round, shuffles the order of agents and then arranges the agents into pairs, so that the order of scheduling is randomized at each time step. Each simulation begins with *N* agents, each initialised with a unique opinion and a value system. Agents’ production of opinions and their value systems evolve according to the model dynamics described in section Model dynamics. Population size was kept constant. Model runs proceeded in discrete time steps, that we call “rounds”. For illustration purposes, Fig 1 shows a flowchart depicting relevant activity during one round.

### Model dynamics

Let *A* = {*a*_1_, *a*_2_, …, *a*_*N*_} be the set of agents in a population, and let *X* = {*x*_1_, *x*_2_, …, *x*_*K*_} be a vector of *K* opinions; each opinion represents a different kind of taste or judgment on a certain topic (e.g., politics, language, religion, music, etc.) and takes its value from the combination of a range of parameters (see Table 1). In the initial state, each agent *a*_*n*_ ∈ *A* is randomly assigned an opinion *x*_*k*_ ∈ *X* selected from *X* without replacement, so that the model is initialised with maximum diversity of opinions. We denote the opinion of an agent *a*_*n*_ at round *r* ∈ {1, 2, …} as *x*_*nk*(*r*)_ ∈ *X*_*r*_. We use *X*_*r*_ = {*x*_1*k*(*r*)_, *x*_2*k*(*r*)_, …, *a*_*Nk*(*r*)_} to denote the vector of opinions in the population at any time *r*. For example, *x*_1*k*(0)_ is the opinion expressed by agent *a*_1_ at round 0, *x*_2*k*(0)_ is the opinion expressed by agent *a*_2_ at round 0, and so on.

**Table 1:** Parameters, state variables and scales.

Model parameters				
Entity	Parameter	Symbol	Number of levels	Value(s)
Agent	Opinion value bias	*β*	11	0.0 to 1.0 in steps of 0.1
	Frequency bias	*β*′	11	0.0 to 1.0 in steps of 0.1
	Memory	*m*	1	3
	Value system of an agent *n*	*S* = {*s*_*n*1(*r*)_, …, *s*_*nK*(*r*)_}		
	Opinion value (assigned by an agent)	*s*		Eq 3
	Agent’s sensitivity to opinion value s	*b*	2	0.0 to 1.0 in steps of 0.1
	Opinion in agent’s memory record	*d*	2	[0, 1]
	Agent’s history	*h*		
	Typical opinion	*x*_*k*_		
	Typical agent	*a*_*n*_		
	Round	*r*		
Global	Initial set of agents	*A* = {*a*_1_, …, *a*_*N*_}		
	Initial number of agents per micro-society	*N*	2	10,100
	Initial vector of opinions	*X* = {*x*_1_, …, *x*_*K*_}		
	Initial number of opinions per population	*K*	2	10, 100
	Number of rounds	*R*	1	100
	Number of games per round	*N*/2	2	5,50
	Institution	*G* = {*g*_1(*r*)_, …, *g*_*K*(*r*)_}		
	Institutional value (assigned by G)	*g*		Eq 1
	Institutional influence	*ε*	11	0.0 to 1.0 in steps of 0.1

Also, let *S* denote a vector of cardinal utility-based values (or value system), which is a probability distribution on X: that is, at each round *r*, any agent *a*_*n*_ has a value system *S*_*n*(*r*)_ = (*s*_*n*1(*r*)_, *s*_*n*2(*r*)_, …, *s*_*nK*(*r*)_). *S*_*n*(*r*)_ assigns a number between 0 and 1 to each opinion in *X*. We denote the value assigned by an agent *a*_*n*_ to an opinion *x*_*k*_ at round *r* as *s*_*nk*(*r*)_ ∈ *S*_*n*(*r*)_. For example, *s*_*n*1(*r*)_ is the value assigned by an agent *a*_*n*_ to opinion *x*_1_ at round *r*, and *s*_*n*2(*r*)_ is the value assigned by an agent *a*_*n*_ to opinion *x*_2_ at round *r*, and so on. *S* evolves according to Eq 3.

At each round, agents are paired randomly. Once agents are paired, they interact by presenting and observing an opinion. Each agent expresses one opinion from *X* (according to the probabilistic function defined in Eq 5. Thus, the vector of opinions *X*_*r*_ contains *N* (one per agent) opinions at each round *r*. After each interaction, agents add both the expressed and observed opinions to their memories. That is to say, at round *r*, when agent *a*_1_ and agent *a*_2_ interact, agent *a*_1_ expresses opinion *x*_1*k*(*r*)_ and agent *a*_2_ expresses opinion *x*_2*k*(*r*)_; both agents store opinions *x*_1*k*(*r*)_ and *x*_2*k*(*r*)_ in their respective memories.

### Model parameters and equations

**Fig 2:**
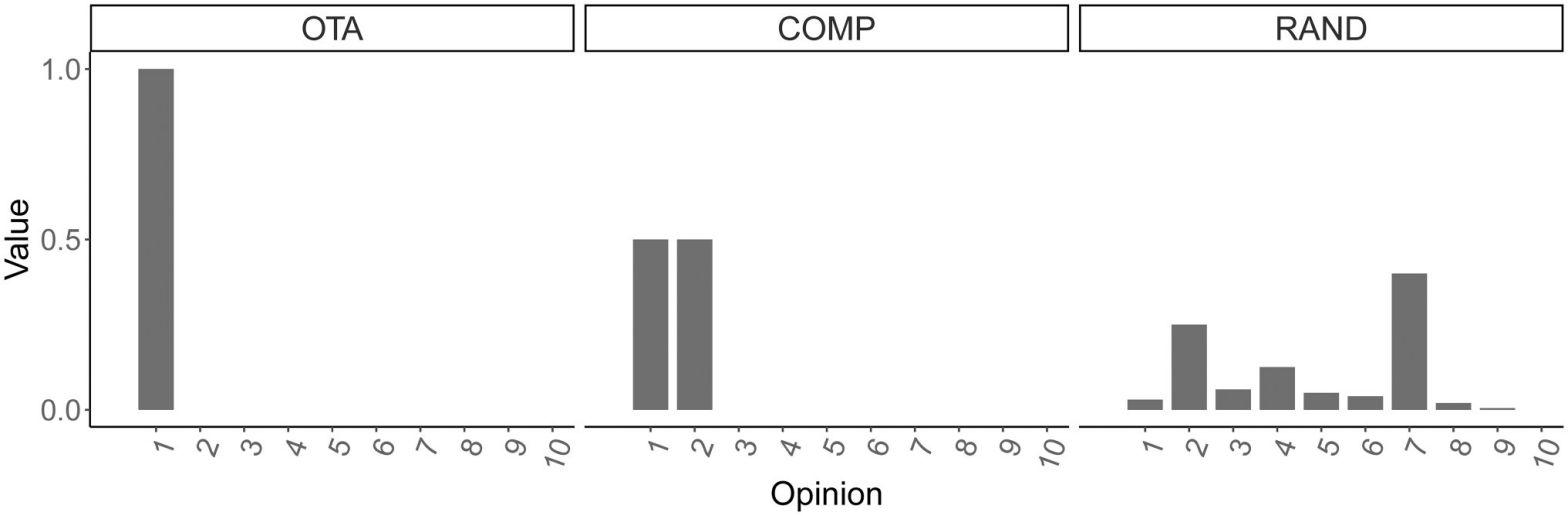
Three example value systems with 10 opinions each illustrating the three types of individual opinion value distribution.

Our model takes the following parameters:

1. Number of agents (*N*): We simulate micro populations of *N* = 10 and *N* = 100 agents. Each agent is initialised with an opinion randomly selected from a pool of *K* = *N* distinct opinions at the beginning of each simulation. We assume that the number of different expressed opinions at each round cannot exceed the number of agents in the population. At each round, each agent expresses exactly one opinion from *X*, so we have that |*X*_*r*_| = *N*.
2. Number of rounds (*R*): Model runs proceeded in 100 rounds. At each round (*r*), the pairing is randomized in such a way that each agent pairs up with another agent (*N*/2 pairs are formed).
3. Value system (*S*): The value system of an agent is a vector of cardinal utility-based values, which is a probability distribution of cardinal preferences on X. It expresses the agent’s preferences about opinions before they have seen any opinion from other agents or from the platform. It is similar to a cardinal utility vector of an agent in the classical rational choice theory [77]. At each round, this vector is then weighted in the probabilistic model by the agent’s opinion value bias (see Eq 5). At the end of each round, the agent’s value system is updated according to Eq 3. We ran simulations in three conditions according to the initial distribution of values allocated to opinions (Fig 2):
  (a) *One Takes All* (OTA). One preferred opinion (*x*_1_) has value = 1, the rest, 0.
  (b) *Competition* (COMP). Two competing opinions (*x*_1_ and *x*_2_) have value 0.5, and the rest, 0.
  (c) *Random* (RAND). We create the initial vector of values by drawing size samples of dimension *N* from a Dirichlet distributed random variable, where *s*_*nk*(*r*)_ ≥ 0 and $\sum_{n=1}^{N}$ *s*_*nk*(*r*)_ = 1.
4. Population homogeneity. We model two conditions:
  (a) In *homogeneous* populations, all agents are initialised with the same value system type.
  (b) In *heterogeneous* populations, the population is divided into two equal sub-populations. Each sub-population is assigned a different type of value system.
5. PR Institution (*G*): The micro-society is governed by a global institution *G*_(*r*)_ = (*g*_1(*r*)_, *g*_2(*r*)_, …, *g*_*K*(*r*)_). *G* is a vector of length *K*, in other words, it contains a value for each opinion. Thus, *g*_*k*(*r*)_ ∈ *G*_(*r*)_ is the value assigned by the institution to opinion *x*_*k*_ at round *r*, for instance *g*_1(*r*)_ is the value assigned by the institution to opinion *x*_1_ at round *r*, and *g*_2(*r*)_ is the value assigned by the institution to opinion *x*_2_ at round *r*, and so on.
  We consider two institutions according to the type of information they aggregate: value-aggregating institutions and opinion-aggregating institutions.
  (i) Value-aggregating institution: This type of institution aggregates value systems proportionally (cardinal-based). At each round *r*, the institutional value *g*_*k*(*r*)_ assigned to each possible opinion-choice *x*_*k*_ is calculated as the arithmetic mean of the *N* values *s*_1*k*(*r*)_, *s*_2*k*(*r*)_, …, *s*_*Nk*(*r*)_, where *s*_1*k*(*r*)_ corresponds to the value assigned by agent *a*_1_ to opinion *x*_*k*_, and *s*_2*k*(*r*)_ corresponds to the value assigned by agent *a*_2_ to opinion *x*_*k*_, and so on. That is, the value assigned by an institution to an opinion *x*_*k*_ at round *r* + 1 is given by the following expression:
    $$\begin{array}{c} g_{k(r+1)}=\frac{1}{N}\sum_{n=1}^{N}s_{nk(r)}=\frac{s_{1k(r)}+s_{2k(r)}+\ldots +s_{Nk(r)}}{N} \end{array}$$ (1)
  (ii) Opinion-aggregating institution: This type of institution directly aggregates the opinions produced by the agents. After agents have expressed their opinions at each round *r*, the value *g*_*k*(*r*+1)_ for a given opinion *x*_*k*_ is calculated as the relative frequency *f* of opinion *x*_*k*_ in *X*_*r*_:
    $$\begin{array}{c} g_{k(r+1)}=f_{x_{k}}=\frac{n_{x_{k}}}{|X_{r}|}=\frac{n_{x_{k}}}{\sum_{x_{k}}n_{x_{k}}} \end{array}$$ (2)
    where n stands for the number of the individual opinions *x*_*k*_ found in *X*_*r*_, and |*X*_*r*_| is the size (number of opinions) in *X*_*r*_.
6. Institutional influence (*ε*): This parameter identifies the extent to which the institution influences agents’ value system. Technically, for each agent *n* and round *r*, if an opinion *x*_*k*_ ∈ *h* (where *h* stands for agent’ history), then the value assigned by an agent *a*_*n*_ to an opinion *x*_*k*_ at round *r* + 1 is a function of the current opinion value *s*_*nk*(*r*)_, the boolean value indicating whether the opinion was expressed in that round by the agent [*x*_*nk*(*r*)_], current institutional value for that opinion *g*_*k*(*r*)_ and institutional influence *ε*.
  In our model, institutional influence is the mathematical complement of the agent’s preference for their own current values and opinions. Agents’ current values and opinions have equal weight in the future value of an opinion. The value assigned by an agent *a*_*n*_ to an opinion *x*_*k*_ is updated at each round according to the following equation:
  $$\begin{array}{c} s_{nk(r+1)}\left(s_{nk(r)},x_{nk(r)},g_{k(r)},\varepsilon \right)=\{\begin{array}{cc} \varepsilon \cdot g_{k(r)}+\left(1-\varepsilon \right)\frac{s_{nk(r)}+\left[x_{nk(r)}\right]}{2} & \text{if}\;x_{k}\in h\\ s_{nk(r)}, & \text{otherwise} \end{array} \end{array}$$ (3)
  where *s*_*nk*(*r*+1)_ stands for the value of opinion *x*_*k*_ at round *r* + 1.
  At each round, [*x*_*nk*(*r*)_] takes the value one if the opinion *x*_*k*_ was expressed in round *r* by agent *a*_*n*_, and takes the value 0 otherwise:
  $$\begin{array}{c} \left[x_{nk(r)}\right]=\{\begin{array}{cc} 1 & \text{if}\;x_{nk(r)}\;\text{is}\;\text{TRUE};\\ 0, & \text{otherwise}. \end{array} \end{array}$$ (4)
  Three conditions of institutional influence were examined in the main analyses:
  (a) *Null Institutional Influence* (*I*_*n*_) (*ε* = 0), where institutions do not influence the evolution of agents’ value systems. This can be thought of as a proxy for a population of agents that is not compliant with institutional values.
  (b) *Moderate Institutional Influence* (*I*_*m*_) (*ε* = 0.5), where institutions have a moderate influence on agents’ value systems but agents also consider their experience and their history of interactions.
  (c) *Strong Institutional Influence* (*I*_*s*_) (*ε* = 1), where institutions have complete influence over the agents. That is, agents are fully compliant with institutional values.
  Two additional conditions were examined to assess the near absence of institutional influence: *I*_005_, where *ε* = 0.05, and *I*_010_, where *ε* = 0.1.
7. Opinion value bias (*β*): identifies the degree of preference for opinions with high value. It encompasses two parameters (*b*, *d*). Parameter *b* is the agent’s sensitivity to opinion value (*s*), and ranges from 0 (not sensitive at all) to 1 (fully sensitive) in steps of 0.1. Parameter *d* specifies whether the opinion is in the agent’s memory record, and equals 1 if the opinion is in memory, and 0 otherwise. Parameter *β* is equal to *b* x *d*. Thus, opinion value bias (*β*) assigns a value from 0 to 1 to each opinion. When opinion value bias is 0, we have a neutral model.
8. Frequency bias (*β*′ = 1 − *β*): This parameter identifies an agent’s preference for opinions that are more frequent in its recent history of interactions. It corresponds to the complement of *β*. This parameter is limited by the agent’s memory size *m*, that is, the maximum amount of history (in rounds) that can influence the opinion choice. We fix *m* to a value of 3 (associated with the best fit in [39, 40]), which means that only opinions observed or produced in the most recent 3 rounds are taken into account for frequency bias. Given an agent *a*_*n*_ and an opinion *x*_*k*_, then *f*(*x*_*k*_∣*h*_*nr*∣*M*, *m*_) is the relative frequency of opinion *x*_*k*_ in the memory of an agent *a*_*n*_ by round *r*. Thus, *f*(*x*_*n*_∣*h*_*nr*∣*M*,3_) corresponds to the relative frequency of opinion *x*_*k*_ in an agent memory for the last 3 rounds.
  For each round in the simulation, for each agent, the model yielded a probability distribution of opinions (*X*_*r*_) for a given history (*h*) of previous rounds, according to the following equation:
  $$\begin{array}{c} \mathrm{Pr}\left(x_{nkr}|h_{nr}\right)=\left(1-\mu \right)\left(1-\beta \right)f\left(x_{k}|h_{nr|M,m}\right)+\left(1-\mu \right)\beta s_{nkr} \end{array}$$ (5)
  where Pr(*x*_*nkr*_∣*h*_*nr*_) is the probability that an agent *a*_*n*_ produces opinion *x*_*k*_ at round *r* given the specific history of agent *a*_*n*_ by round *r*. For each parameter combination examined we ran 2000 simulations.

### Quantifying and categorising opinion diversity

We quantify the diversity of opinions in the population using Shannon’s entropy. In order to facilitate the comparison with other metrics and to eliminate the effect of different population sizes and time series, we normalise entropy by log_2_ *N* to obtain *H*_*N*_(*X*_*r*_)∈[0, 1]:

$$\begin{array}{c} H_{N}\left(X_{r}\right)=-\sum_{x_{i}\in X_{r}}\frac{p\left(x_{i}\right)\log_{2}p\left(x_{i}\right)}{\log_{2}N} \end{array}$$ (6)

where *X*_*r*_ corresponds to the set of opinions at round *r*, *p*(*x*_*i*_) is the probability of the *i*^th^ opinion in that set, and *N* is the number of opinions (which is equal to the number of agents). High entropy corresponds to high diversity and low convergence on shared opinions.

We consider the vector of opinions at each round (*X*_*r*_) as a proxy for a one-dimensional opinion spectrum (e.g. the left–right political spectrum). That is, we consider that the position of opinions in *X*_*r*_ represents their degree of extremism. Opinions located at the extremes of the vector are considered extreme, while opinions located in the centre of the vector are considered moderate. We categorise the equilibria, that is, the long-term stabilised ratios of the competing opinions, in five separate groups:

1. Extreme consensus (*E*): all the agents in the population converge on a shared opinion which is either on the extreme left or on the extreme right of the opinion axis.
2. Moderate consensus (*M*): all agents converge on a shared opinion which is neither on the extreme left nor on the extreme right of the opinion axis.
3. Polarisation (*P*): more than 45% of the population converges on an extreme opinion and more than 45% of the population converges on the opposite extreme opinion.
4. Diversity (*D*): three or more opinions have attracted at least 10% of the population.
5. Indetermination (*I*): all other equilibria. These are situations in which there is neither a strong consensus nor extreme polarisation nor a great diversity of opinions. In general, these are situations where a majority and a minority opinion coexist.

## Results

This section reports the results of opinion dynamics simulations under the influence of value-aggregating and opinion-aggregating institutions. First, we analyse the evolution of the diversity of expressed opinions for each of the combinations of institutional influence, initial value systems and homogeneity. Then we explore how our model behaves in the absence and near absence of institutional influence. Next, we assess the robustness of our findings with respect to population size constraints. Finally, we look at the opinion categories formed when the frequency of different opinions stabilises.

### Opinion diversity over time

For these simulations, agents were initially assigned an opinion selected from a pool of opinions without replacement. Since we start off with maximum diversity, we expect diversity (entropy) to decrease over generations. We will compare diversity decreases against neutral baselines in which *β* = 0. These cases are neutral or unbiased because the value of the preferred opinion (*s*) in the value system is multiplied by an opinion value bias (*β*) of 0, and therefore the results are identical for all three value systems (*OTA*, *COMP* and *RAND*). Any deviations from the neutral models are due to the effects of changing the level of the values of the parametric model. Simulation outcomes show that the co-evolutionary processes of institutional influence, value systems and individual biases implemented in the model tend to stabilise the diversity of opinions over time.

#### Value-aggregating institution

**Fig 3:**
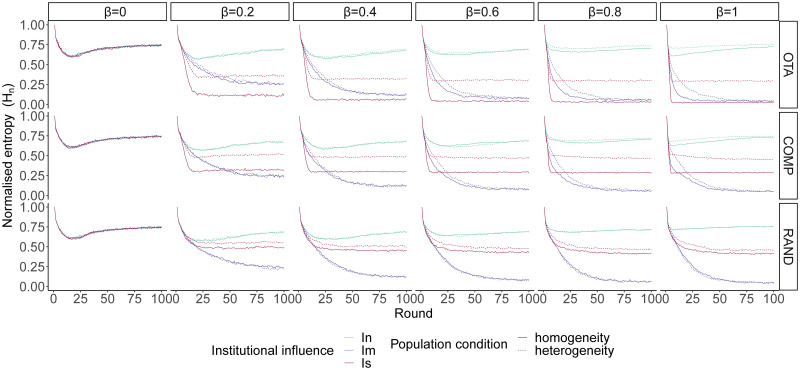
Opinion diversity (measured as normalised Shannon entropy) under value-aggregating institutions, averaged over each level of institutional influence (*I*_*n*_ = neutral; *I*_*m*_ = moderate and *I*_*s*_ = strong), population homogeneity or heterogeneity and opinion value bias (*β*) under three types of value systems: *OTA* (one takes all), *COMP* (competition between two opinions) and *RAND* (random). Agents tend to converge on a shared opinion when institutional influence is moderate. Under strong institutional influence the equilibrium tends to reflect the initial diversity of agents’ value systems in the population. Convergence on shared opinions accelerates as *β* increases.

We first consider a value-aggregating institution that is able to optimally aggregate agents’ value systems (or cardinal utility vectors). Fig 3 shows that convergence on a shared opinion is highly likely in the medium to long term (before 100 rounds) when institutional influence is moderate and agents’ bias towards opinion value is moderate to high (*β* > 0.2). The consistency and robustness of this result across conditions is striking, although initial conditions with scattered distributions of value systems (*RAND*) and heterogeneous populations tend to slightly slow down the convergence process on a shared convention. PR institutions under moderate influence act as a reference point for convergence on a shared opinion by providing the necessary conditions for positive feedback that generates global coordination. PR institutions are initially diverse, as they represent the diversity of values of the population. Gradually, agents accumulate small deviations towards one of the main opinions. This in turn generates institutions that are more and more focused on a single opinion. Finally, the institution ends up representing a single opinion, which stabilizes the convention process.

When institutional influence is strong, stabilisation of opinion dynamics tends to reflect the initial diversity of agents’ value systems in the population. Under *OTA*, opinion frequencies tend to settle down to a stable state with one successful opinion (*H*_*n*_ ≈ 0) in the case of homogeneous populations and two main competing opinions (*H*_*n*_ ≈ 0.3) in heterogeneous populations. All other opinions have a negligible small frequency at equilibrium. Under *COMP* a coexistence stable state is reached at *H*_*n*_ ≈ 0.3 in the homogeneous condition and at *H*_*n*_ ≈ 0.5 in the heterogeneous condition, while under *RAND* the stable state is reached at around *H*_*n*_ ≈ 0.5 with heterogeneous populations exhibiting slightly higher entropy than homogeneous populations. In all these scenarios, institutional influence is so strong that opinion dynamics always orbit around the opinions promoted by the emerging institution. Since PR institutions proportionally reflect the value systems of the agents, the population ends up finding its equilibrium of opinions around the initial priors of the population.

Opinion value bias tends to accelerate convergence on shared opinions across conditions. Nevertheless, our results show that the effect of the institutional influence is very robust and qualitatively similar for all levels of biased social learning examined (*β* > 0.2). On the other hand, with unbiased social learning (*β* = 0) neutral selection leads to an equilibrium at *H*_*n*_ ≈ 0.75, that is, below the maximum diversity of the system. This is because drift causes some opinions to disappear before stabilisation.

#### Opinion-aggregating institution

We now consider a model in which the emergent institution directly aggregates the opinions expressed by the agents. All other conditions were exactly the same as in the simulations with value-aggregating institutions.

**Fig 4:**
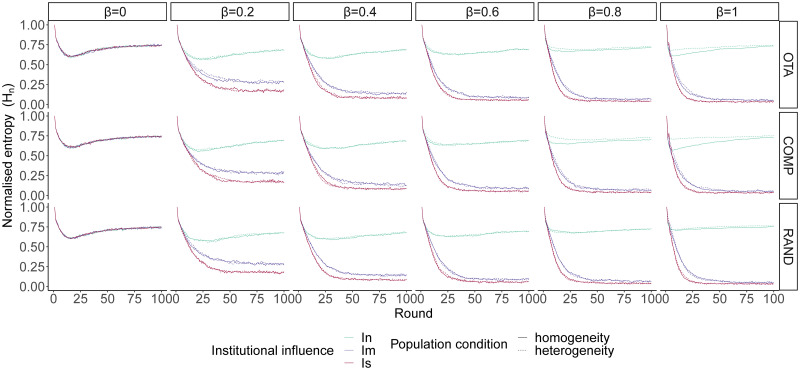
Opinion diversity (measured as normalised Shannon entropy) under opinion-aggregating institutions, averaged over each level of institutional influence (*I*_*n*_ = neutral; *I*_*m*_ = moderate and *I*_*s*_ = strong), population homogeneity or heterogeneity and opinion value bias (*β*) under three types of value systems: *OTA* (one takes all), *COMP* (competition between two opinions) and *RAND* (random). Agents tend to converge on a shared opinion when institutional influence is moderate or strong. Under strong institutional influence convergence occurs at a faster rate. Convergence on shared opinions accelerates as *β* increases.

As seen in Fig 4, when institutional influence is moderate or strong and agents are biased towards opinions with high value, the population tends to converge on a shared opinion irrespective of the level of population homogeneity and initial diversity of values in the population. This result is robust across conditions. The system is initiated with maximum entropy (one opinion per agent). Accordingly, the emerging institutions in the first rounds are diverse because they aggregate proportionally the opinion expressed by each agent. Gradually, as the system evolves, the the most frequently expressed opinions tend to be those most valued within agents’ value systems.

Interestingly, unlike under the framework of value-aggregating institutions, what we now have with opinion-aggregating institutions is that both moderate influence and strong influence tend to make the population converge on a shared opinion. This is because under institutions that aggregate value systems, deviations towards one of the opinions do not occur as frequently as under institutions that aggregate the opinions expressed at each point in time. Opinions expressed change faster than the underlying value systems, therefore, when institutional influence is strong, the value-aggregating system tends to converge stably around the value systems (which may hold one, two or several competing opinions), but the opinion-aggregating system is more volatile, allowing the system to move more quickly away from the population’s original value systems and eventually converge on shared opinions. This suggests that under strong institutional influence, the type of information that the institution aggregates is crucial, with higher levels of convergence the lower the stability of the aggregated information. In other words, opinion-aggregating institutions with strong influence facilitate convergence because, paradoxically, by proportionally reflecting the opinions of the population they make it easier for agents to move away from their original value systems.

### No (and very low) institutional influence

We collected data from simulations of populations where there was little or no institutional influence. The aim was to study in detail the behaviour of our model when mechanisms facilitating global coordination are non-existent or close to zero. Under these conditions, we can analyse the impact that the scarcity of institutional incentives has on the spontaneous emergence of conventions in the population, as well as investigate whether the emergence of global consensus is possible in the absence or quasi-absence of institutions.

**Fig 5:**
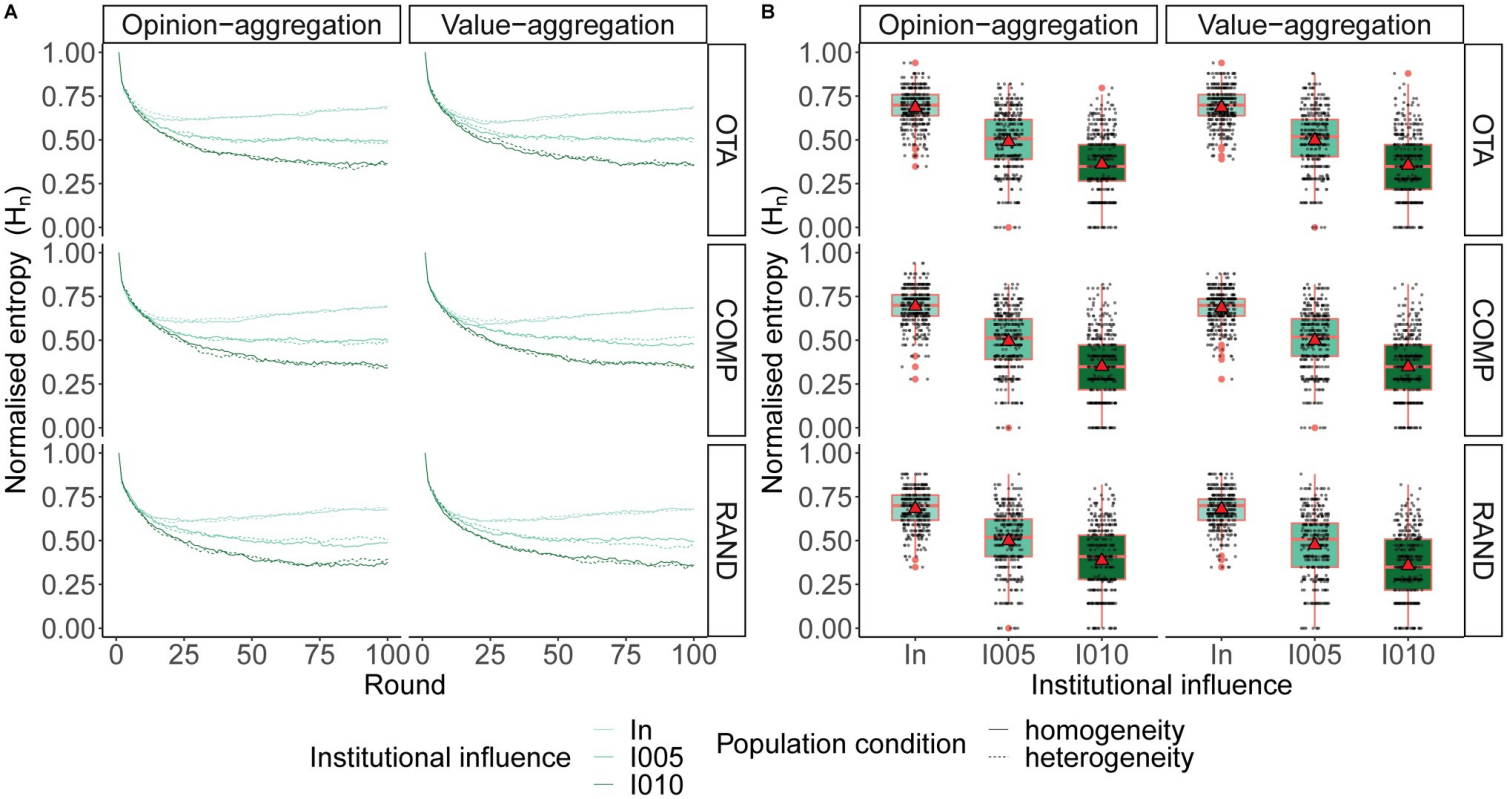
A. Opinion diversity (measured as normalised Shannon Entropy) for *I*_*n*_ (*ε* = 0), *I*_005_ (*ε* = 0.05) and *I*_010_ (*ε* = 0.1), under opinion-aggregating and value-aggregating institutions, averaged over each level of population homogeneity and initial value system when opinion value bias (*β* = 0.5). B. Standard box plot of opinions diversity at round 100 for *I*_*n*_ (*ε* = 0), *I*_005_ (*ε* = 0.05) and *I*_010_ (*ε* = 0.1), under opinion-aggregating and value-aggregating institutions, averaged over each initial value system. Red line indicates median, red triangle indicates mean. Each black dot represents the final entropy of a simulation in that condition.

Fig 5 shows that, in contexts of low institutional incentives (i.e. in the range of 0 < *ε* < 0.1), slight increases in institutional influence produce sharp drops in the diversity of opinions. Populations under *I*_*n*_ find their average equilibrium around *H*_*n*_ = 0.68, with 0% of the simulations reaching global consensus and 0% of the simulations reaching convergence below *H*_*n*_ = 0.25 after 2000 simulations (for simulations with an intermediate *β*). However, an increase of only 0.05 percentage points in institutional influence leads to *H*_*n*_ = 0.49, with 1.54% of the simulations reaching global consensus and 7.45% of the simulations reaching convergences below *H*_*n*_ = 0.25. Moving up from *I*_005_ = 0.05 to *I*_010_ = 0.1 leads to *H*_*n*_ = 0.36, with 6.08% of the simulations reaching global consensus and 25.79% of the simulations reaching convergences below *H*_*n*_ = 0.25.

### Assumptions concerning population size

Our model makes a crucial assumption about the society in which agents interact and exchange opinions over time. While we have shown that the institutional impact of PR institutions affect the diversity of opinions over time under certain cognitive conditions in 10-agent micro-societies, it is important to assess the robustness of these findings. Here, we run simulations with 100 agents to test the influence of population size on model outcomes.

**Fig 6:**
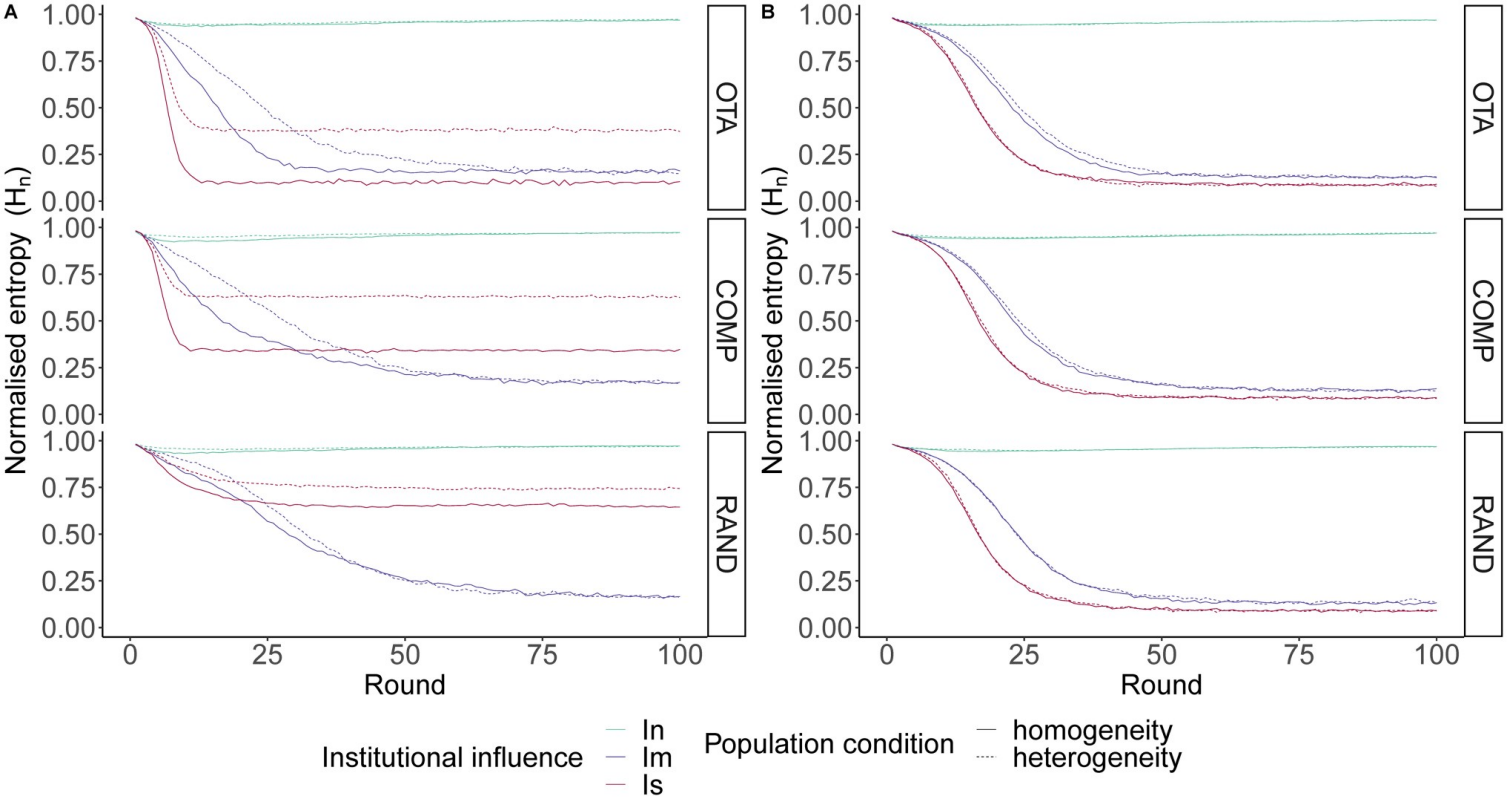
Diversity of opinions in 100-agent micro-societies, averaged over each level of institutional influence, level of population homogeneity and value system at *β* = 0.5. A: Value-aggregating institution; B: Opinion-aggregating institution. Results from these controlled simulations are qualitatively similar to the results presented above using 10-agent micro-societies.

Our simulations of 100-agent micro-societies under value-aggregating institutions and opinion-aggregating institutions show that the effects of homogeneity, institutional influence and value systems are qualitatively similar to the runs using 10-agent micro-societies (Fig 6). Under value-aggregating institutions, moderate institutional influence always leads to high levels of convergence, while convergence under strong institutional influence is dependent on the initial value systems. On the other hand, in the case of opinion-aggregating institutions, both strong and moderate institutional influence lead to high levels of convergence, and strong influence accelerates convergence compared to moderate influence. There are, however, two visible differences in the evolution of opinion dynamics in the larger population. First, in the absence of institutional influence *I*_*n*_ (*ε* = 0), agents’ limiting opinion (i.e. the final vector of opinions in the population) tends to reproduce the initial state of opinion in the population. While in small populations a certain convergence was observed due to drift, here this effect is marginal. Second, the fall of entropy in the larger populations resembles a sigmoid function. This is particularly true in cases where institutional influence is moderate *I*_*m*_ (*ε* = 0.5) and there is high initial dispersion in the population’s value systems (*RAND*). In these scenarios, the initial opinion diversity persists for longer in larger populations. Gradually, the process of convergence in the local interactions increases the slope of the curve of entropy decline. Then, entropy goes to 0 as time → ∞. These parameter settings only increase the frequency of surviving opinions, but all other conclusions hold.

### Opinion categories at equilibrium

Finally, we unpack the patterns of consensus, polarisation and diversity observed when the frequency of opinions stabilises in the model. We first analyse the results of the simulations with value-aggregating institutions and then those of the simulations with opinion-aggregating institutions.

#### Value-aggregating institution

**Fig 7:**
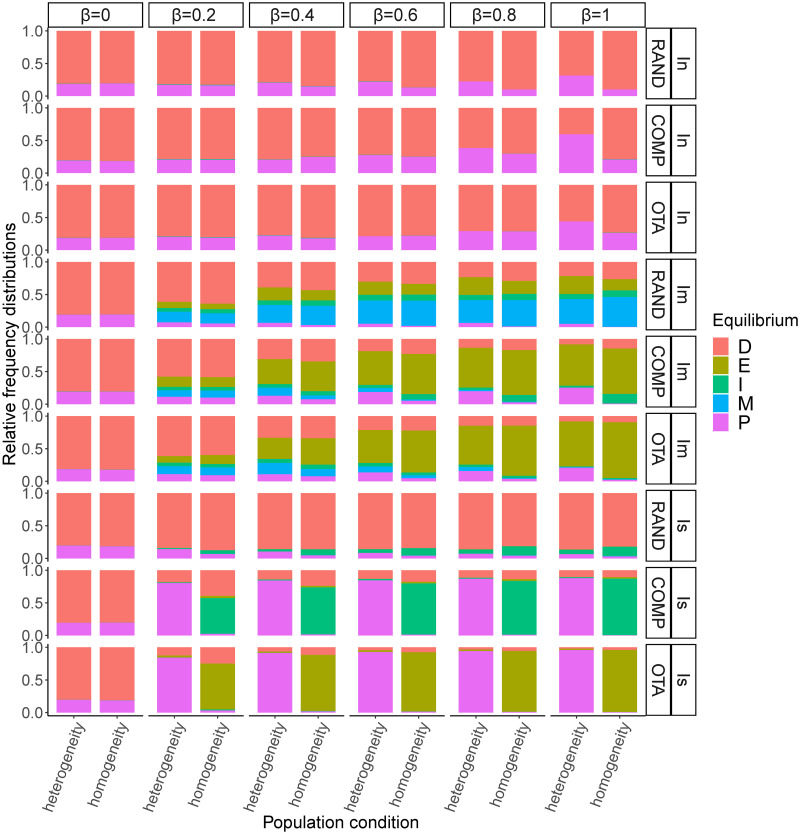
Relative frequencies of each equilibrium category when the frequency of opinions stabilises in the model under value-aggregating institutions. Institution influence: *I*_*n*_ = neutral; *I*_*m*_ = moderate and *I*_*s*_ = strong; *β* = opinion value bias; value system types: *OTA* = one takes all, *COMP* = competition between two opinions, *RAND* = random. When institutional influence is intermediate, moderate consensus is more likely when the initial dispersion of values is high *RAND*, and extreme consensus is more likely under *OTA* and *COMP*. When institutional influence is strong, the probability of polarisation increases. In general, in the absence of institutional influence there is a high diversity of opinions at equilibrium.

Our simulations show that the intensity of institutional influence determines the type of equilibria (Fig 7). As observed above, the probability of reaching a consensus is greater under moderate institutional influence. The analysis of opinion categories at equilibrium shows that the combination of institutions with moderate influence and *OTA* or *COMP* value systems leads to *extreme consensus*. On the contrary, under *RAND* value systems there is a high probability of *moderate consensus*. This means that greater initial variation in the population’s value systems works as a deterrent to convergence onto extreme views.

While no institutional influence generally allows for high levels of diversity of opinions in the population, when institutional influence is strong equilibrium is determined by the initial distribution of value systems in the population. Under strong institutional influence, random value systems (*RAND*) result in high diversity of opinions, while one-takes-all (*OTA*) leads homogeneous populations to *extreme consensus* and heterogeneous populations to *polarisation*. *COMP* tends to form two opinion clusters at equilibrium, with *polarisation* in heterogeneous populations and *indetermination* in homogeneous populations.

Our results are similar in some respects to those of previous simulations of opinion dynamics on platforms that make personalised recommendations [30]. Extremism of agents’ limiting opinion is non-monotonic in platform’s influence, and extreme opinions at equilibrium (*extreme consensus* and *polarisation*) are less likely when the initial value systems and opinions are more balanced. However, there are also fundamental differences in the type of institution and process that the two models simulate. In our case, the simulation of proportional representation institutions shows that *extreme consensus* is likely when platform’s influence is intermediate (although a *moderate consensus* is more likely under *RAND*). *Polarisation* in two opinion clusters is more likely under strong institutional influence. On the other hand, null institutional influence tends to lead to high diversity of agents’ limiting opinion in our model (with biased learning and heterogeneity increasing the probability of polarisation).

#### Opinion-aggregating institution

**Fig 8:**
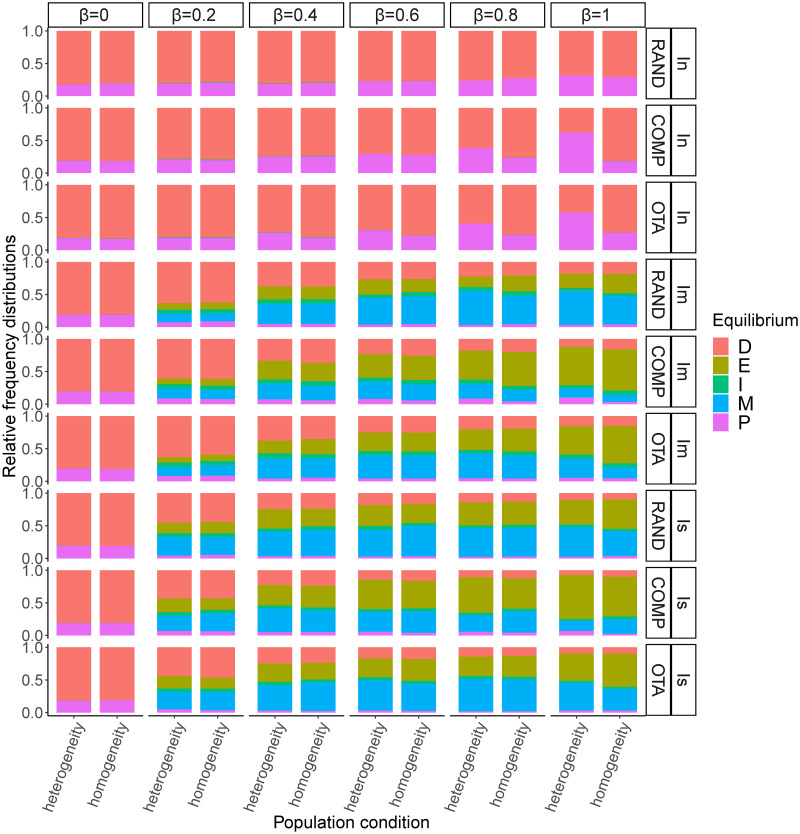
Relative frequencies of each equilibrium category when the frequency of opinions stabilises in the model under opinion-aggregating institutions. Institution influence: *I*_*n*_ = neutral; *I*_*m*_ = moderate and *I*_*s*_ = strong; *β* = opinion value bias; value system types: *OTA* = one takes all, *COMP* = competition between two opinions, *RAND* = random. When institutional influence is intermediate, moderate consensus is more likely when the initial dispersion of values is high *RAND*, and extreme consensus is more likely under *OTA* and *COMP*. When institutional influence is strong, the probability of polarisation increases. In general, in the absence of institutional influence there is a high diversity of opinions at equilibrium.

The picture changes when institutions directly aggregate the opinions expressed by agents (Fig 8). When institutional influence is moderate, greater variation in initial value systems (*RAND*) remains the best way to favour the formation of a *moderate consensus*. *OTA* and *COMP* continue to be associated with a high frequency of *extreme consensus*, however, when comparing with value-aggregating institutions, we observe that the equilibria has shifted in favour of *moderate consensus*, to the detriment of *polarisation* and, above all, extreme consensus. This is particularly true for intermediate levels of *β*.

When institutional influence is strong, the situation is similar to that of moderate institutional influence, with all conditions leading to either *moderate* or *extreme* consensus in biased learning populations. *RAND* and *OTA* yield a higher frequency of *moderate consensus* than *COMP*.

Interestingly, for strong institutional influence, the equilibria we find under opinion-aggregating institutions are very different from those under value-aggregating institutions. In all cases, the equilibrium has shifted towards consensus. Polarisation (which was the norm in heterogeneous populations with *OTA* and *COMP*) and diversity (in *RAND*), have turned into consensus, whether extreme or moderate. The explanation for this lies in the type of information that institutions aggregate. Opinions change rapidly (faster than value systems), which facilitates the formation of institutions that reflect values far removed from the original cardinal values in the population. This promotes value change in the population and at the same time facilitates the possibility of convergence in the local interactions of agents. Gradually, deviations towards one of the opinions (either extreme or moderate) produce institutions more and more focused on a particular point of the spectrum of views, which favours the emergence of global conventions.

### Spatial domains of opinion categories

**Fig 9:**
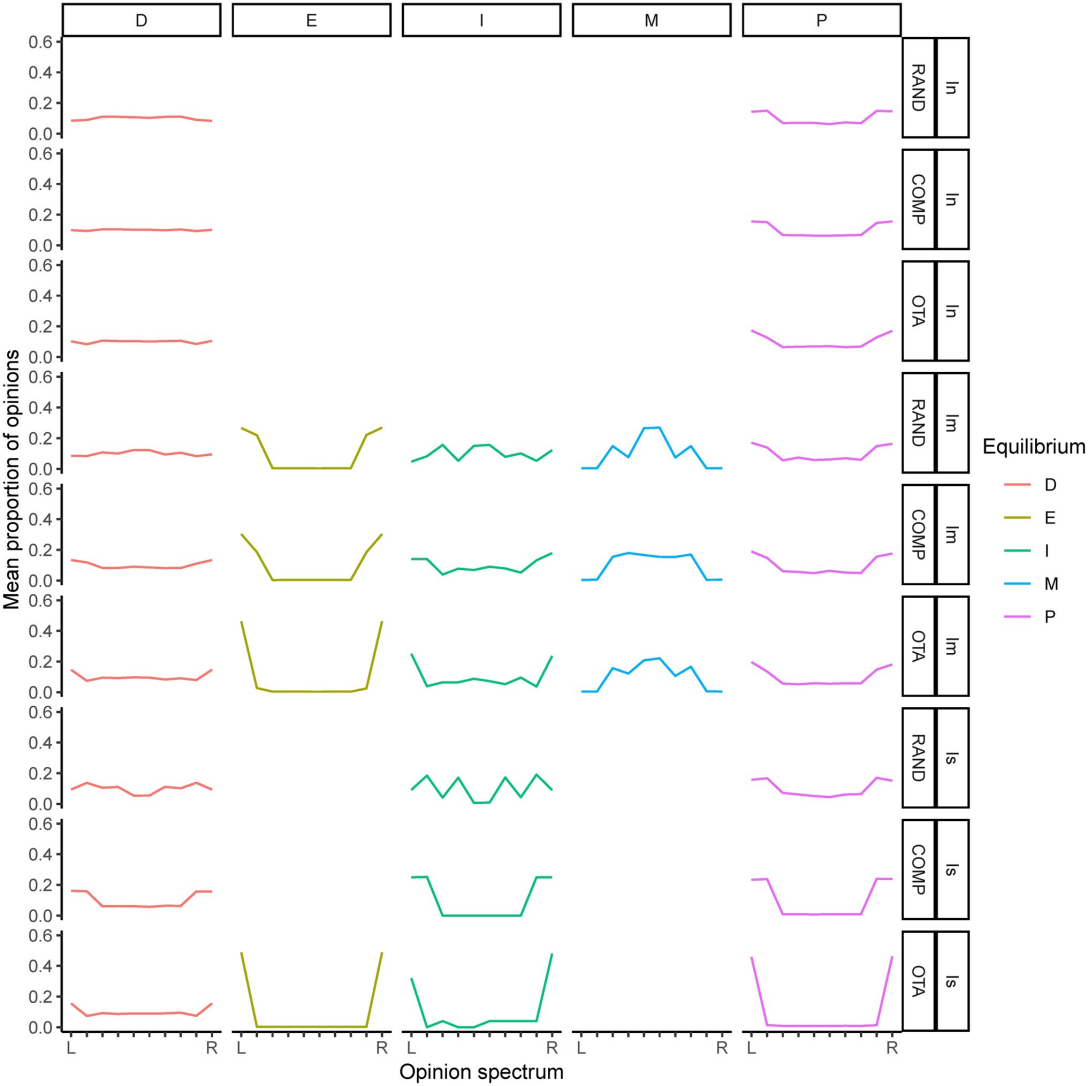
Mean proportion of opinions under value-aggregating institutions in each type of opinion equilibrium by condition after 2000 simulations. We exclude low frequency events (which may reduce the reliability of the opinion distribution and lead to higher margin of error) by removing any output that occurs less than 0.05%.

**Fig 10:**
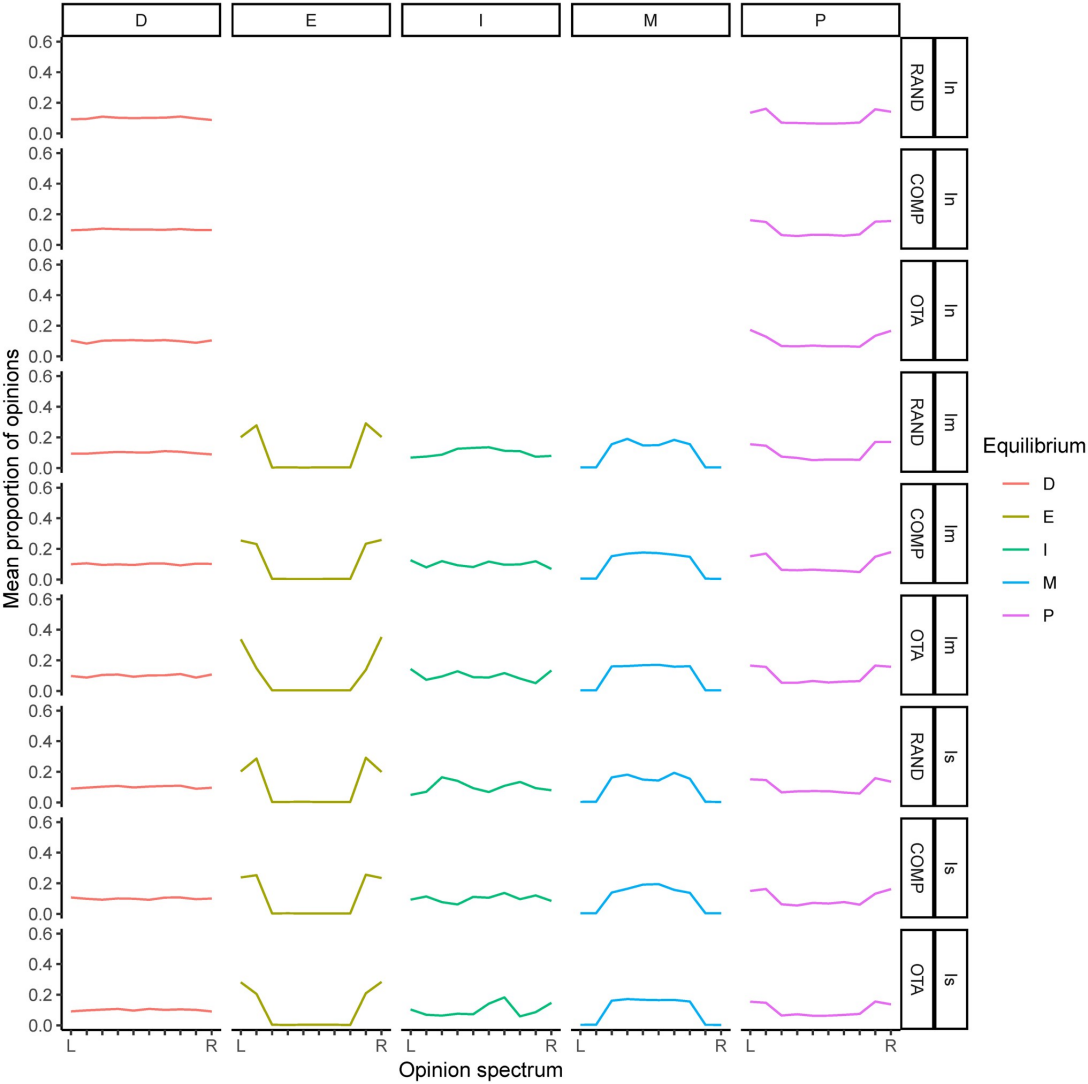
Mean proportion of opinions under opinion-aggregating institutions in each type of opinion equilibrium by condition after 2000 simulations. We exclude low frequency events (which may reduce the reliability of the opinion distribution and lead to higher margin of error) by removing any output that occurs less than 0.05%.

This section shows visualisations of the long-term ratios of the competing opinions in a spectrum from extreme left to extreme right, grouped by opinion category, institutional influence and initial value system. Figs 9 and 10 show each opinion category associated to its spatial domain for value-aggregating and opinion-aggregating institutions, respectively. In particular, we show the average relative frequency of opinions on the spectrum of opinions at equilibrium after 2000 simulations. Each of the distributions can be thought of as the proportion of votes (or opinions in a poll) in favour of each political option after two thousand independent electoral processes, all after prolonged deliberation until the stabilisation of the opinion ratios. The analysis is intended to illustrate the quantitative meaning of the spatial dispersion of opinion in the equilibrium of each condition.

We can observe that the (algorithmically value-aggregated) opinion distributions exhibit a shape that more closely reflects the original value systems. A paradigmatic case can be found under strong institutional influence and *OTA*: Whereas under value-aggregating institutions the cases of polarisation tend to focus on the more extreme views at each end of the spectrum (reflecting an initial *OTA* distribution), under opinion-aggregating institutions the cases of polarisation can be found in the range of the two most extreme views (implying a deviation from the initial *OTA*). In other words, the polarised equilibrium under opinion-aggregating institutions has shifted away from the initial value systems. Similarly, we can observe that under value-aggregating institutions *extreme consensus* is concentrated at one end of the opinion spectrum (reflecting the initial value system of the population), while under opinion-aggregating institutions *extreme consensus* occurs at either end of the spectrum (again, implying a departure from the initial value systems).

As expected, *moderate consensus* has higher frequencies in the central opinions of the spectrum, with a higher statistical dispersion in the conditions where *moderate consensus* is the most frequent equilibrium. On the other hand, distributions representing the category labelled *diversity* have a wide dispersion across the spectrum of opinions. As for the category labelled *indetermination*, which corresponds to the complement of *D*∪*E*∪*M*∪*P*, we note that it takes on more “exotic” shapes. This category generally corresponds to situations where there is neither a clear consensus nor a great diversity of opinion in the equilibrium. In general, it occurs when there is a majority opinion coexisting with a minority one. Under value-aggregating institutions, *indetermination* tends to look more like (but not consolidate as) *extreme consensus*. Under opinion-aggregating institutions, *indetermination* tends to become more dispersed, resembling the *diversity* category. Interpretation of this category should therefore be made with caution. Its impact on the results shown above is limited, as it is not very frequent. It should be noted however that it is the most frequent equilibrium in the case of homogeneous populations under value-aggregating institutions with strong institutional influence and *COMP*. In this case, the most plausible interpretation is that *indetermination* means extreme quasi-consensus.

## Discussion

We have examined how opinion dynamics evolve in a social network where interactions are mediated by the influence of PR institutions. We used an ABM where institutions and value systems are synchronised in an iterative process. In particular, we looked at the conditions under which convergence on shared opinions can be obtained.

### General remarks

Our methods diverge from the previous literature on opinion dynamics and institutional influence by creating a system in which agents’ value systems and institutions are synchronised. Moreover, opinion dynamics are based on a mean-field feedback rule that is not constant but evolves as a reflexive signal that endogenously aggregates agents’ opinions and value systems. Unlike research on opinion dynamics using differential equations approaches, we use a multilevel-selection, agent-based model. This approach allowed us to detect the strength of selection at any organizational level in the simulated platform.

Our model is useful for exploring opinion dynamics in social media platforms that use recommendation algorithms with a vocation for the proportional representation of preferences and opinions. This way, the model helps add new scenarios of exploration to the formal theories of emergence of social conventions and institutions.

We find that:

1. Intermediate institutional influence leads to consensus: extreme or moderate. Institutions with moderate influence function as a benchmark for agents beliefs, which facilitates global coordination. The best conditions for achieving a moderate consensus are provided by a combination of high initial diversity of value systems and institutions with moderate influence on agents’ opinions.
2. Strong institutional influence can lead to polarisation. This is particularly true in heterogeneous populations under value-aggregating institutions. Under opinion-aggregating institutions the most common equilibria are moderate and extreme consensus.
3. Null institutional influence leads to higher diversity of opinions in the population. However, the combination of highly biased agents and heterogeneous populations can lead to polarisation.
4. According to our simulations, the best way to avoid extremism is to increase the initial diversity of value systems in the population.

Comparisons between our model and previous differential-equation-based models are limited due to the differences in the simulated microcosms [30, 33]. Yet it may be useful to pay attention to some key aspects where the results of our models agree: (i) when initial views are more balanced, there is a low probability of extreme consensus, and (ii) when the platform’s influence is strong, there is a higher probability of polarisation on two extreme views (although in our model, polarisation is highly dependent on population structure and the type of aggregated information). These findings suggest that diversity may prevent extremism across platforms with different aggregation rules and recommendation algorithms. However, studies using real-world data are needed to confirm this hypothesis.

We have described a model where institutions affect agents’ value systems through their direct influences on agents’ opinions. This argues against the axiom of exogenous preferences: that is, the idea that individual’s preferences come from outside the model and are unexplained by the model. By contrast, we model endogenous preferences, or preferences that are shaped by individual’s internal responses to the external state of affairs. In this way, we have proposed a simple model, rooted in the tradition of the field of cultural evolution, that helps to formalise the basic mechanics of opinion dynamics under the assumptions of Bowles’s hypothesis of institutional influence [36].

### In the absence and quasi-absence of institutions

We examined convergence in the absence and quasi-absence of institutions. As we explained above, there is a growing interest among scientists in explaining under what conditions shared conventions can emerge spontaneously in the absence of institutional incentives. Most social and cognitive scientists assume that institutional mechanisms can explain global coordination [69–72, 78]. However, social evolutionary theories argue that, under specific circumstances, global conventions can emerge from local interactions [73, 74, 76]. In particular, using an experimental approach, Centola & Baronchelli [75] demonstrated how simple changes in a population’s network structure can lead to the emergence of global conventions without the need of institutions in place to guide the process.

In our model, local coordination alone in the total absence of institutional influence does not lead to convergence on a shared opinion. Given these seemingly contradictory accounts, it is important to note that:

i Our model generally agrees with theories and studies suggesting that institutions facilitate the formation of conventions. However, comparisons between theories and experiments on the emergence of social conventions should be taken with caution, given the enormous variability in the nature of the instances of conventions used in different studies (e.g. drawings, words, opinions, etc…), which may lead to faulty generalisations.
ii Our model does not suggest that spontaneous conventions are not possible without institutions, and in fact, it is consistent with the findings of Centola & Baronchelli [75]. In their well-known 2014 study, Centola & Baronchelli found that homogeneously mixing population was the only condition in which a global consensus emerges. On the contrary, in the spatial network and random network trials, the most popular convention in most trials was well bellow 40%. This is exactly what we found in our simulations, whose network structure was randomised.
iii While in most previous studies individuals were rewarded for coordinating locally, in our model agents have no specific incentive to do this. The social scenarios we simulate involve neither the immediate specification of players’ strategy spaces nor fixed and symmetric reward functions. Instead, they involve a set of initial preferences over opinions that are unique to each agent and evolve over time under the influence of institutions, choice, experience and agents’ deviations from neutrality (biases).
iv Interestingly, in some of the conditions, our model yields relatively high levels of convergence for very low—though not zero—levels of institutional influence. This suggests that very low levels of institutional influence may be sufficient to generate a positive feedback loop that favours the emergence of global conventions from local interactions.

An interesting direction for future research using our model would be to investigate new paradigms that empirically test how people value opinions dynamically. This approach could check whether and/or how specific ways of evaluating opinions affect opinion dynamics. Also, an interesting possibility not considered in this study is to explore how simple changes in the population’s network structure can lead to the formation of spontaneous social conventions. This could be used for model calibration with previous experimental studies.

### Opinion aggregation as a catalyst for value change and convergence

One of the main findings of the present study is that the nature of aggregated information matters when it comes to explaining opinion dynamics. In particular, we have shown that, under strong institutional influence, the opinion-aggregation rule used for the formation of institutions facilitates values shift. On the contrary, the value-aggregation rule preserves the initial value systems. In a social scenario where opinions change rapidly, if the only aggregated information is the opinions expressed by the agents, then the emerging institutions will generally be far from reflecting the underlying value systems of the agents. Therefore, institutional influence will promote a change of values in society. At the same time, institutions will gradually incorporate deviations towards those opinions that are proportionally more frequent, which, together with the weakening of the agents’ initial values, generates a positive feedback that facilitates global coordination on a shared opinion.

The idea that the aggregation of individual inputs on collective outputs affects the outcome of the system is at the core of social choice theory [79, 80]. However, researchers have often focus solely on the emergence of collective behavior from repeated decentralized interaction and neglect the existence of aggregating institutions [81]. In this study we have formalised the effects of proportionality by using two simple aggregation rules based on information of different nature (i.e. opinions and preferences). In doing so, we have shown that the nature of aggregated information matters when it comes to explaining the emergence of social conventions. This finding has implications for understanding information propagation, crowd behaviour, social cognition and collaborative learning. For example, verification of our findings would reveal that an aggregation system that implements a first-past-the-post rule may be more conducive to reaching new consensus, while an aggregation system based on evaluative voting (cardinal preferences) would be more conducive as a pre-established order maintenance strategy. The exploration of these hypotheses may be of interest to researchers working on questions related to collective intelligence including how humans perform best in groups or how to organise people in business and politics in ways that optimise information sharing and decision making. Our model can be used in the future to explore alternative aggregation rules and their impact on opinion dynamics.

### Limitations and other avenues for future research

A limitation of our model is that we use a discrete-time approach to continuous-time contagion dynamics. As described in [82], discretising time as a proxy for continuous-time dynamic processes can lead to a restriction on the values of the model parameters that can accurately be studied. Also, our model makes a crucial assumption about how agents assign value to opinions by equating the weight of the experience-based value system and the current opinion in the future value of an opinion.

Future research could test our results against real data obtained in an experiment that mimics the logic behind our model. Calibrating the model with real data would allow us to find the parametrisation that best fits the experimental opinion dynamics. An efficient method of carrying out this calibration is based on the use of genetic algorithms as fitness functions [83].

Another interesting avenue for future research would be the collection of actual data from social media platforms that implement the principles of proportional representation to aggregate information and display it back to users. Unfortunately, it is not easy to find current social media platforms that use this type of algorithm. However, the development of projects focused on the reconstruction of the plurality of a country’s political landscape from Twitter data (see [6]) could be the basis for future efforts to explore the effect of plural institutions on opinion dynamics. Such a project could help us better understand how the behaviour of users exposed to information aggregated proportionally differs from that of users of mainstream platforms.

## Conclusion

To conclude, what we present here is a simple model that allows us to examine opinion dynamics under different scenarios of institutional influence, where platforms make “opinion-based” and “cardinal utility value-based” proportional recommendations. We found that, in certain regions of the parameter space, an increase in the initial diversity of value systems acts as a deterrent of extremism. We also showed that moderate institutional influence can lead to moderate consensus and strong institutional influence can lead to polarisation. On the other hand, opinion aggregation works as a catalyst for value change and convergence. In our model, randomised networks with local coordination alone (in the absence of institutional incentives) do not lead to convergence on shared opinions, but very low levels of institutional influence are sufficient to generate global conventions.

Our model can be of interest to researchers investigating the effects of social media platforms and institutions on opinion dynamics and the emergence of social conventions. In particular, the model is useful to inform about how the interactions between institutional influence, value systems and individual biases affect the emergence of shared opinions in human populations.
